# Due deference to denialism: explaining ordinary people’s rejection of established scientific findings

**DOI:** 10.1007/s11229-017-1477-x

**Published:** 2017-06-30

**Authors:** Neil Levy

**Affiliations:** 1 Macquarie University, Sydney, Australia; 2 University of Oxford, Oxford, UK

**Keywords:** Climate change, Deference, Irrationality, Belief, Psychology

## Abstract

There is a robust scientific consensus concerning climate change and evolution. But many people reject these expert views, in favour of beliefs that are strongly at variance with the evidence. It is tempting to try to explain these beliefs by reference to ignorance or irrationality, but those who reject the expert view seem often to be no worse informed or any less rational than the majority of those who accept it. It is also tempting to try to explain these beliefs by reference to epistemic overconfidence. However, this kind of overconfidence is apparently ubiquitous, so by itself it cannot explain the difference between those who accept and those who reject expert views. Instead, I will suggest that the difference is in important part explained by differential patterns of epistemic deference, and these patterns, in turn, are explained by the cues that we use to filter testimony. We rely on cues of benevolence and competence to distinguish reliable from unreliable testifiers, but when debates become deeply politicized, asserting a claim may itself constitute signalling lack of reliability.

## Introduction

According to a recent Gallup poll (Newport 2014), around half of the population of the United States reject the theory of evolution. Many ordinary people reject one or more of the well-established central tenets of anthropogenic global warming (AGW): that global warming is occurring; that it is very significantly the product of human activity, and that it is a serious problem (Gass 2015). These issues are uncontroversial among those with the relevant expertise, and the vast majority of those who reject the expert consensus have no special skills or sources of evidence that would justify their rejection of the expert consensus. Why do substantial numbers of people nevertheless reject the views of the experts on these topics?

In this paper, I will argue that a large part of the explanation lies in the cues that we utilize in deciding what weight to put on testimony. While we are apt to accept testimony—to defer to others—we reject testimony from sources that signal unreliability by evincing cues of incompetence or lack of benevolence. When science becomes politicized, expression of the scientific consensus may itself come to serve as a signal of lack of benevolence to those on one side of the issue, leading to rejection of the testimony. On all sides, filtering mechanisms may be working as designed, but for reasons beyond the purview of the individuals involved, warrant may accrue to one side alone.

In the first section of this paper, I will briefly sketch an intuitive rival explanation why ordinary people come to embrace views at odds with those of the relevant experts: because they are misinformed or irrational. A deficit of information may play a limited role in explaining dissent, but neither a deficit of information nor of rationality provides anything like a full explanation. In the next two sections, I begin to lay the groundwork for my own explanation. I argue, first, that the well-known weaknesses of human reasoning actually equip us to be effective collective deliberators, and that science owes its epistemic success to the way in which it harnesses and potentiates this deliberation. Collective deliberation, properly constrained and guided, is our best method for discovering significant truths. But the features that make it so powerful are not apparent to individuals. Despite being adapted for collective deliberation, we are epistemic individualists, disposed to overvalue our own individual deliberation relative to that of groups. In fact, we are epistemic individualists *because* we are adapted for collective deliberation.

Under many conditions, epistemic individualism is able to play its role of enhancing collective deliberation only if it is appropriately constrained by a disposition to defer to others, such that the results of collective deliberation come to be accepted. These facts together play an important role in explaining dissent. Because we are epistemic individualists, we may place too little weight on the outcome of properly constituted group deliberation, which is in fact our best method of tracking truth. The disposition to defer can rescue us from our individualism, but deference is sensitive to certain cues that signal trustworthiness; in particular cues of reliability and benevolence. The politicization of science may produce circumstances in which the content of scientific claims triggers the filters on epistemic deference, by signalling a lack of trustworthiness.

## Deficit accounts of dissent

A highly intuitive explanation of how non-experts come to embrace beliefs that conflict with those of the scientific consensus turns on a deficit on the part of dissenters: either a *rationality* or a *knowledge* deficit (or both). Those who reject evolution (for instance) might be badly misinformed, not only about the evidence supporting the theory but even about its basic claims. Many dissenters are misinformed or irrational, of course. But there is no systematic relation between scientific knowledge or rationality, on the one hand, and dissent, on the other. Neither knowledge nor rationality predicts the positions people take on these debates. Rather, it is their political allegiances (and especially support of free market ideology) that are predictive (McCright and Dunlap 2011; Schuldt et al. 2011; Lewandowsky et al. 2013a, b).

Climate sceptics typically greatly underestimate the proportion of scientists who accept the central claims of AGW (Leiserowitz et al. 2014; van der Linden et al. 2015; Cook et al. 2016). They are apt to claim that there is no consensus. However, those sceptics with higher scientific literacy are quite good at identifying what climate scientists believe about, say, the effects of carbon dioxide, or about the relationship between climate change and severity of hurricanes, and those who are more likely to reject the consensus are not more likely to get these questions wrong (Kahan 2015). Deficits in knowledge play only a limited role in explaining dissent.

Deficits in reasoning skills fare worse than deficits in knowledge in explaining dissent. While liberals who are better informed, exhibit higher levels of scientific literacy and are disposed to be more reflective are more likely to accept the views of relevant experts than those who are less well informed, have lower levels of scientific literacy and are less reflective, the reverse is true of conservatives: more information and better reasoning skills correlate with *lower* rates of acceptance of the expert consensus (Kahan 2015). Conservatives who score higher on items in the Ordinary Science Intelligence scale which specifically measure dispositions to override intuition in favour of reflection have higher rates of rejecting the consensus view (Kahan 2015). It is not a lack of a capacity to engage in this kind of reasoning or an unwillingness to deploy it that explains resistance to the scientific consensus among this group.

Indeed, conservatives may deploy their reasoning skills to *defend *the views to which they are committed. Reflective processes are often utilized to rationalize our intuitive judgments rather than correct them (Mercier and Sperber 2011). The capacity to develop alternative explanations for observations or to spot weaknesses in arguments enables conservatives more effectively to discount evidence they are motivated to reject (Kahan 2015). Since those who score higher on Cognitive Reflection Test items have a greater capacity to identify or invent flaws in arguments that conflict with their antecedent commitments, they may see the balance of the evidence as *more* strongly in their favour than less able conservatives, and therefore come to hold more strongly anti-consensus views. On average, conservatives who reject the expert view on global warming or on evolution do not reason any worse than liberals who accept it. In fact, those who reject the consensus most strongly may be on average *better* informed and more capable reasoners than those who accept the consensus.

While ignorance may play a role explaining dissent (van der Linden et al. 2015, 2017), it cannot explain the dissent of the knowledgeable. I will suggest that patterns of deference to testimony explain why some individuals reject the claims of particular experts. In the next two sections, I provide evidence that collective deliberation is central to the pursuit of truth. Subsequently, I argue that collective deliberation requires deference to testimony, but that deference is sensitive to cues of reliability that play an important role in explaining the rejection of scientific expertise.

## Cognition disciplined

Collective deliberation is often normatively superior to individual. On a variety of reasoning tasks, individuals perform surprisingly badly. Performance on the Wason selection task is a familiar example. Participants are asked to identify which of four cards need to be turned over to test whether a simple conditional (if *p* then *q*) is true. One side of each card represents either *p* or $\sim$*p* while the other represents either *q* or $\sim$*q*. Around 10% of participants select all and only the right cards when the test is presented in its standard form (Evans et al. 1993).^1^ We are also motivated reasoners. We scrutinize claims we are motivated to reject much more carefully than those we are motivated to accept (Lord et al. 1979; Kunda 1990; Ditto and Lopez 1992; Lord and Taylor 2009).^2^ These weaknesses appear surprising, given that reasoning well is adaptive. However, these weaknesses may be strengths in the context of collective deliberation (Mercier and Sperber 2011).

Under some conditions group reasoning is more powerful—better able to identify significant truths—than individual reasoning. Under these conditions, groups not only outperform the average individual; they may do significantly better than the best individual within the group. When groups of deliberators are allowed to exchange reasons for their choices, they do very much better on the Wason task; performance rises from around 10% correct to 70% (Mercier et al. 2015a). Transcripts of exchanges within groups indicate that the correct answer is accepted as a result of arguments. The benefits of group over individual cognition appear to be cross-culturally robust (Mercier et al. 2015b). Multi-agent simulations provide confirmatory evidence that the exchange of arguments is a powerful means of tracking truth (Betz 2013). Moreover, the superiority of the group over the individual does not require that one member has the right answer prior to deliberation: group deliberation may enable the aggregation of the genuine insights of several members and the rejection of the false hypotheses of some of the same individuals. This is an instance of the *assembly bonus effect* (Collins and Guetzkow 1964), in which groups perform better than the best individuals that compose them.

Some of the pathologies to which individual reasoning is subject are explained if they are adaptations for group deliberation. Biased assimilation, the disposition to become more strongly attached to antecedent views given genuinely mixed evidence (rather than moderating them, which would seem to be the rational response to evidence some of which genuinely supports our view and some of which conflicts with it), appears to be the product of the asymmetrical deployment of reasoning capacities, with considerable effort devoted to rejecting arguments for conclusions that are unfavourable to us. Group deliberation harnesses this asymmetry (see Betz 2013 for supportive simulations). If a diversity of views is held by members of the group, a broader range of conflicting arguments comes under rational scrutiny: none get a free pass; accordingly, diverse groups are less vulnerable to polarization (Fishkin and Luskin 1999). A diversity of opinion coupled with a disposition toward motivated reasoning contributes to “an efficient form of *division of cognitive labor*” (Mercier and Sperber 2011: 65).^3^

However, group deliberation seems to be vulnerable to pathologies of its own. There is evidence that it sometimes leads to *group polarization *(Isenberg 1986). Group polarization occurs when deliberation causes a group to become more extreme in its views. The mechanisms of group polarization may be rational, but they nevertheless sometimes cause the group to move further away from the truth (Sunstein 2002). However, the risk of group polarization may be greatly reduced. Becoming more extreme arises from the asymmetrical scrutiny of arguments for and against the conclusions antecedently held by group members (Betz 2013). If the group is sufficiently diverse in its initial opinions, scrutiny would not be systematically biased in this way and polarization may be avoided. The spectacular success of science is, I suggest, partially explained by the way in which scientific institutions harness the power of group deliberation while minimizing the risks of the pathologies it is subject to.

## Science as group cognition institutionalized

While the capacity for group deliberation may play an important role in explaining the unrivalled human capacity to discover significant truths, it does not explain the spectacular growth in our capacity to understand, make accurate predictions about, and intervene into the world around us since the seventeenth century. An important part of the explanation for this historically novel development, I suggest, is that the scientific revolution involved the *institutionalization* of group deliberation.

Group deliberation is most successful when certain conditions are satisfied. Condorcet’s jury theorem shows that as groups grow in size, they will converge on the truth, on the assumption that each ‘voter’ has a greater than 0.5 probability of choosing the correct option and makes his or her choice independent of the others. Obviously, these conditions are idealizations that are rarely or never satisfied in the real world. Nevertheless, the jury theorem may hold under more general assumptions (Ladha 1992; Hahn et al. 2016). But even these more minimal conditions may go unsatisfied. Groups may lack sufficient diversity. Charismatic leaders may, advertently or not, suppress dissenting opinions, or mechanisms of informational cascade may lead to groupthink. When members are led to think that the majority of group members hold an opinion at variance with theirs, they may self-silence. Low status individuals may feel unable to express their views, or their opinions may be given little credibility (Fricker 2007). Ridicule powerfully silences dissent (see Sunstein 2005 for evidence of many of the ways in which groups may fail to take advantage of information their members possess). Science, when it is working well, has mechanisms to ensure diversity and sufficient independence.

For instance, the anonymous reviewing of research findings may play a significant role in allowing lower status individuals to be heard. Peer review ensures that risking rejection for publication does not entail risking ridicule or social rejection: the knowledge that a paper is rejected is confidential (if reviewing is triple anonymous, not even the editor need know). Anonymizing of manuscripts also minimizes the impact of authors’ status on how their claims are perceived. Anonymity also enables (possibly junior) reviewers to express opinions without fear of reprisal.

Science also institutionalizes rewards for successful dissent.^4^ Rewards are greatest for those who overturn accepted findings, whereas smaller advances within the framework of accepted views (while of course exponentially more common) are not as well rewarded.^5^ Science also allows group cognition to work at multiple levels at once. Scientific research is conducted by groups of individuals, often with different disciplinary perspectives and different skills. This ensures that results submitted for publication have already been scrutinized from a variety of perspectives and embody the results of group cognition that has been structured to enhance its veritistic features. Peer review increases the range of people who assess the methodology and argumentation, bringing in further independent perspectives. If the paper is published, it becomes accessible to a much broader range of researchers, in many different parts of the world, each with different disciplinary perspectives and different background beliefs. Post-publication, group deliberation is potentiated, by ensuring that the scrutiny of research is not merely by groups, but groups of groups (say the social scientists of North America).

At the same time as the institutionalization of science enhances the veristic features of group deliberation, it reduces those features that might lead away from truth. Groups with shared biases may amplify them (Sunstein 2005). By exposing claims to scrutiny by geographically and intellectually diverse groups, a range of competing biases is brought to bear. Groups of individuals who are strangers to one another are better at tracking truths than groups of individuals who have a shared history (Solomon 2006). In premodern times, groups of deliberating strangers were very rare. The institutionalization of science ensures that what once was rare is now commonplace.

Many of the features I have pointed to postdate the initial growth of science. It was only after the scientific takeoff that there was a critical mass of researchers to allow for the institutionalization of these procedures. The role played by the learned societies in the development of science is crucial to its initial takeoff. Societies like the ‘invisible college’ that surrounded Robert Boyle took the first steps toward institutionalizing debate and the exchange and criticism of ideas. Science is a story of increasing institutionalization of group deliberation and thereby potentiation of its powers.^6^

## Epistemic individualism

The success of science at institutionalizing group deliberation apparently makes dissent by non-experts more mysterious. A scientific consensus is the product of group deliberation at its best, and is therefore exponentially better justified than rival views in favour of which ordinary people dissent. Part (but only) part of the explanation, I argue, is that we are epistemic individualists, who attach more value to the product of our individual deliberation than is warranted.

Ordinary people and experts alike appear unaware that when certain (relatively undemanding) conditions are satisfied, group deliberation is much more reliable than individual cognition.^7^ Mercier et al. (2015a) had individuals in six different groups estimate individual and group success at the Wason selection task. Groups were composed of individuals with experience of managing teams, experts in the psychology of reasoning, Indian participants and Japanese participants, as well as Western undergraduates. In five of the six, participants greatly overestimated individual success. In some conditions, participants performed the task and were then provided with an explanation of the correct response. This debiasing procedure lowered their estimate of individual success, but it continued to be much higher than actual performance. Across all six studies, participants greatly underestimated the relative benefits of group deliberation, even subsequent to solving the problem in groups. The only group of participants who accurately estimated individual performance was psychologists with expertise in the psychology of reasoning. Since the dismal performance on the Wason selection task by individuals is common knowledge in the field, this accurate estimate is unsurprising. This group of participants nevertheless underestimated group performance to the same extent as some groups of laypeople. All groups vastly underestimated the benefits of group deliberation relative to individual deliberation. Epistemic individualism appears to hold across cultures. Indian and Japanese subjects seem to exhibit the same pattern of overestimation of individual reasoning relative to group deliberation (Mercier et al. 2015a). Japanese subjects show much the same pattern of improvement in reasoning in group settings (Mercier et al. 2015b) and Mayan subjects exhibit the same pathologies of individual reasoning but improvement in collective contexts (Castelain et al. 2016).

If we owe our epistemic success, in important part, to group deliberation, our epistemic individualism is puzzling. We noted earlier that many of the pathologies of individual reasoning can be explained if they are in fact adaptations for collective deliberation. I speculate that the same is true for our epistemic individualism: somewhat paradoxically, we are disposed to discount collective deliberation because this disposition enhances collective deliberation. Overconfidence in one’s own reasoning may lead individuals to seriously consider and argue for novel explanations for observations for which there is already an accepted explanation, limiting the influence of informational cascades or mechanisms that suppress dissent. Epistemic individualism may bring about sufficient independence of ‘votes’ for group deliberation to be truth-conducive.^8^

Given the widespread overvaluation of individual reasoning relative to group deliberation, individuals who reject the scientific consensus might do so, in part, because they judge that they are as well placed to evaluate the evidence (for climate change, or for the safety and efficacy of vaccines, say) as the scientists who form the consensus. They might prefer their own assessment over that of the experts, because they (rightly) take the latter to be the product of collective deliberation, while holding (wrongly) that individual deliberation outperforms or is at least as powerful as collective.^9^

Just *because* epistemic individualism is so prevalent, however, it cannot by itself constitute the explanation for partisan dissent from the scientific consensus. Epistemic individualism may explain why dissenters are untroubled by their dissent and why they look for alternative explanations of phenomena that have already been adequately explained. But there is no evidence that dissenters are more inclined to epistemic individualism than others. If epistemic individualism is an adaptation for collective deliberation, we ought to expect it to be widely distributed. We are all epistemic individualists, but we are not all climate change deniers or creationists. On most topics, most of us accept the claims of the relevant experts. Despite our epistemic individualism, we defer. Explaining the partisan pattern of acceptance and dissent requires us to understand the way in which we filter testimony.

## Epistemic deference

If epistemic individualism is an adaptation for collective deliberation, there must be some mechanism whereby the products of collective deliberation come to be accepted. Under many conditions at least, epistemic diversity must be ‘transient’ (Zollman 2010), and that, in turn, requires that we are apt to defer to others.

Deference is in fact ubiquitous.^10^ Global warming ‘skeptics’ do not come to their beliefs unaided. Instead, they defer to others (Kahan 2015; Keller 2015). Conversely, liberals exhibit much higher levels of trust in science (Lewandowsky and Oberauer 2016). As a consequence, conservatives and liberals are disposed toward biased assimilation of evidence supporting and opposing their views (Corner et al. 2012). In part, biased assimilation is caused by asymmetrical deference: accepting views from some sources and not others.

Asymmetrical deference may be partially explained by reference to message content (Keller 2015). Because responding to AGW very likely requires limitations on free markets, it is highly unpalatable to strong supporters of markets. While ‘solution aversion’ (Campbell and Kay 2014) may explain some of the observed variance, conservative distrust of environmentalism (and support for unfettered markets) appears to arise from, rather than cause, the partisan split. Conservationism has historically been a strong current within conservative thought, and up until recently there was no partisan divide on the environment, either within congress or on the part of the general public (McCright et al. 2014).

While message content certainly influences the disposition to defer (Harris 2012), sensitivity to properties of the testifier sometimes trumps content. Israelis and Palestinians evaluated a peace proposal significantly more or less favorably depending upon whether it was presented as coming from Palestinian negotiators or Israelis (Maoz et al. 2002). Similarly, conservative and liberal attitudes to proposals for welfare policies were much more strongly influenced by whether the proposals were said to be supported by Democrats or Republicans than by the content of the proposals (Cohen 2003). In fact, sensitivity to properties of testifiers is able to trump content even when the content of the testimony is disagreeable to hearers. Corrections of false claims are effective when they come from sources that share the ideology of the hearer (Nyhan and Reifler 2013) and also when they come from sources that can be expected to find the claim they affirm contrary to their own ideological interests (Berinsky 2017). Thus, corrections of false claims about Obamacare, for instance, are effective for both conservatives and liberals when they come from conservatives; the former because they share the ideology of the source, and the latter because they know that the source is likely to find the claim unpalatable. These data are powerful evidence that content-based explanations are insufficient on their own to explain deference.

This sensitivity to testimony source emerges early in childhood (Clément 2010; Sperber et al. 2010). From a very young age, children show a preference for informants who show signs of being benevolent and competent; scrutiny of informers intensifies around the age of four (Mascaro and Sperber 2009). I suggest that this filter plays a significant role in explaining the different patterns of deference exhibited by conservatives and liberals. The disposition to defer to others with whom we share a political or religious outlook is continuous with the disposition to use benevolence as a cue to reliability; we are disposed to see those with whom we share a political outlook and/or a religious affiliation as those who are benevolent toward us and our interests (or, perhaps, the disposition to use benevolence as a cue to reliability is just a special case of a disposition to defer to those with whom we share values). The use of political and religious affiliation as a proxy for benevolence is adaptive, inasmuch as sharing our values is being disposed to promote the things we value and to oppose the things we disvalue. The use of competence as a proxy for reliability protects us against fools; the use of benevolence as a proxy protects us against exploitation (see Hahn et al. 2016 for a model of how update on testimony is normatively rational as a function of perceived reliability and benevolence of sources).

Both sides utilitze these proxies to filter testimony. Thus, conservative Americans come to their anti-consensus views on these topics in precisely the same way in which their liberal counterparts come to their views: by deferring to those they rightly take to be more knowledgeable than, as well as benevolent toward, them. But because the topic has come to be politicized, this disposition to defer ensures that they do not defer to (or their chains of deference will not bottom out in) groups of scientists who espouse a view contrary to theirs. Since opposition to AGW has come to function as a marker of group allegiance, affirming the ‘wrong’ view *constitutes* a signal of a lack of benevolence and thereby of reliability. That is, since the left has come to be identified strongly with a particular view on AGW, affirming that view is signalling support of a political set of values and thereby a lack of benevolence to conservatives.

Whereas for liberals chains of deference trace back to the relevant scientific experts, and therefore to properly constituted collective deliberation, conservatives’ chains of deference end in ‘merchants of doubt’ (Oreskes and Conway 2010), or maverick scientists. Conservatives do not defer to scientists, or to their think-tank intermediaries or more local representatives, because while these sources exhibit cues of competence they fail to pass tests for benevolence. Liberals are epistemically luckier: they are disposed to defer to the most competent individuals and institutions, because these individuals and institutions pass tests for benevolence as well as for competence. Liberals defer to sufficiently large groups of sufficiently expert deliberators to ensure that their beliefs have a high degree of warrant; conservatives defer to a much smaller group of genuine experts and their chains of deference trace back as much or more to non-experts.^11^ These facts (which are outside the purview of the individuals at the end of each chain) entail that one set of beliefs is very much better warranted than the other. Biased assimilation may thus be individually rational, whether it leads toward better or worse warranted beliefs.^12^

It should be emphasised that there is nothing about the mechanism that undermines the warrant of conservatives’ beliefs about climate change or evolution that entails liberal invulnerability to it. Liberal trust in science is currently very much higher than that of conservatives, reflecting a loss of trust in science by conservatives since the 1970s (Gauchat 2012). Parallel historical processes could undermine liberal trust in science. Such an erosion would leave liberals vulnerable to individually rational but unreliable epistemic deference.^13^ At the present time, however, dissent from the scientific consensus seems more often to be seen from those who strongly endorse free market ideology (there are, however, two important issues on which dissent is bipartisan: GMOs and vaccination; Lewandowsky et al. 2013b). Interestingly, the negative correlation between endorsement of free market ideology and rejection of climate change is significantly stronger than the negative correlation between the first and rejection of other well-established facts, such as the fact that smoking causes lung cancer and that HIV causes AIDS (Lewandowsky et al. 2013a). That suggests that content-based mechanisms play an independent role in epistemic deference. Whether the conservative suspicion toward science generalizes to other domains of expertise is currently unknown.

It is a further, interesting, question how issues come to be politicised such that espousing the warranted view constitutes a signal of unreliability. One live possibility is that the merchants of doubt, driven by specific commercial interests, undertook a successful strategy of politicising climate science such that it would come to serve as a marker of political affiliation and thereby a cue for benevolence or its lack. A deliberate campaign aimed at identifying AGW with the political and social left may have brought about a situation in which espousing an attitude on the topic constitutes evincing a strong signal of political affiliation and thereby of possessing or lacking reliability. This hypothesis is empirically tractable, though to my knowledge the detailed historical work required to support it remains to be done.

## Conclusion

I have suggested that we are disposed to be epistemic individualists not despite but *because* we are reliant on collective cognition: epistemic individualism is an adaptation for group deliberation. Under a variety of circumstances, epistemic individualism must be appropriately constrained for it to be adaptive for group deliberation: our disposition to defer to those with the appropriate markers of competence and benevolence allows epistemic individualism to be epistemically virtuous.^14^

When cues to reliability dissociate from genuine expertise, epistemic individualism may not be appropriately constrained, despite our continuing to defer. When societies become polarized and matters of fact deeply politicized, our dispositions to defer may produce systematic divergences on matters of fact. This is not an individual failing. Not only are the conservatives who reject climate science neither less rational nor always less well-informed than the liberals who accept it, the mechanisms that dispose them to defer to benevolent and competent individuals are working as designed. It is further facts, about earlier links in the chain of deference, that entail that their beliefs are unwarranted, not facts about how they deploy their cognitive resources.

There is a broader moral here. With regard to scientific questions (where a great deal of expertise is required to distinguish signal from noise), deference is warrant-preserving when it is supported by appropriate cultural institutions or structures. It is not enough that the science be institutionalized; its dissemination to non-experts, too, depends on appropriate cultural structures. Those who reject the consensus may be no less epistemically virtuous or conscientious than those who accept it. Their misfortune is to find themselves on the end of chains of transmission that are not warrant-providing. Ensuring that most of us find ourselves at the end of chains of deference that trace back to sufficiently large groups of sufficiently expert deliberators requires attention to the social structures in which we are embedded, rather than to the deficits and strengths of individual cognizers.

## Footnotes

1 Performance is significantly better when the rule tested concerns cheating in social contracts (Cosmides and Tooby 1992) or failing to follow precautionary rules (Fiddick et al. 2000), despite the fact that the logical form of these problems is identical to that of the standard form. The content-sensitivity of performance has formed a central plank of arguments for the claim that there are dedicated modules in the mind.

2
Jern et al. (2014) demonstrate that the processes that lead to biased assimilation of evidence may be cool and rational, rather than driven by affect or irrational.

3 Mercier and Sperber (2011) and Sperber et al. (2010) attribute this division of labor to the confirmation bias (Nickerson 1998). However, Sharot and Garrett (2016) present evidence that people update on their priors in ways that depart from Bayesian rationality only when testing a claim unfavourable to them, rather than one that is consistent with their prior beliefs or hypotheses. This suggests that the division of cognitive labor requires that some individuals find some of the hypotheses under discussion uncongenial (of course, we may care about a hypothesis for many reasons, including the fact that we have staked something on its being true).

4
Kitcher (1993) and Goldman (1999) have each presented groundbreaking work analysing how the pursuit of credit in science can lead to a near optimal distribution of research strategies in science under plausible conditions.

5 These claims are subject to an important qualification. While normal science incentivizes dissent *within* what Kuhn famously called the paradigm, it discourages dissent *from* the paradigm. A scientist who rejects evolution tout court, for instance, must be prepared to pay a reputational cost. Scientists are also educated in ways that discourage dissent from the central features of reigning paradigms: a scientist who produces results that conflict with it is likely to suspect her methodology or her observations, rather than the paradigm itself. Whether we should see this selective but central discouragement of dissent as a necessary cost of good science or instead as itself epistemically virtuous is an open question. However, acceptance of a paradigm does not prevent the accumulation of anomalies, which (should they ever become sufficiently weighty) might lead to the upending of the paradigm. Anomalies are few and far between with regard to the general outlines of climate science, evolution, the efficacy of vaccines, and other foci of dissent.

6 Of course, we shouldn’t idealize science. As the current reproducibility crisis within social psychology (and beyond) illustrates, there are also incentives at work that decrease the reliability of truth tracking. Top journals, for instance, often put a premium on novelty and it is difficult to find a publication venue for replications or failed replications. That reduces the extent to which research findings are adequately scrutinized. However, the relevant institutions are taking steps to increase the reliability of research, including preregistration of hypotheses and an analysis plan, which reduces researcher degree of freedom and therefore minimizes the possibility of (advertently or not) manufacturing a significant finding. The Open Science Foundation’s reproducibility project institutionalizes replication and scrutiny by multiple independent groups. The recent failed attempt to replicate ego depletion, for instance, involved independent replication attempts at two dozen labs, in different parts of the world. The failures to which science has been subject have arisen from an insufficient exposure of findings to group deliberation, and there are signs that some of these failures are being addressed.

7 Building on classic work by Rozenblit and Keil (2002), Sloman and Fernbach (2017) show that people attribute knowledge to themselves when *other* members of their epistemic community possess the relevant understanding. This evidence demonstrates that ordinary people *implicitly* recognize the social nature of knowledge production. But the knowledge they self-attribute is individual: when they are asked to provide details, they significantly lower their confidence in their knowledge (if they recognized that they possessed knowledge as nodes in a social network, they would not expect themselves to be able to provide such details and their failure would not decrease their confidence in their knowledge possession). In fact, implicit recognition of the social distribution of knowledge may play a role in the generation of epistemic individualism: if people did not recognize that there is a sense in which they genuinely possess knowledge as nodes in a network, they would not overinflate the degree to which they possess individual knowledge. I thank a reviewer for this journal for calling my attention to this literature. The reviewer reminds me of something I had known but failed to recall; a decade before Sloman and Fernbach I myself claimed that epistemic individualism might have its roots in the illusion of explanatory depth (Levy 2007).

8 Overconfidence in individual reasoning is unlikely to be adaptive across all circumstances. Sharot and Garrett (2016) suggest that overconfidence is adaptive in environments in which costs are not significantly greater than possible benefits, and speculate that the disposition might therefore be sensitive to features of the environment. Overconfidence might therefore be a facultative adaptation to relatively benign environments.

9 A google search for “groupthink”; “scientists”, and “climate change” returns about 127,000 results; a survey of the top 20 hits indicates that about two thirds of them are devoted to decrying the ‘groupthink’ of climate scientists (with most of the rest devoted to analysing the tactic of crying ‘groupthink’).

10 The ubiquity of deference has only recently come to be recognized in epistemology; the recent explosion of work on testimony is itself testament to the recognition of how great a role it plays in our epistemic lives. Coady (1992) is the seminal work; Lackey and Sosa (2006) presents more recent developments.

11 Perhaps climate sceptics’ chains of deference do not trace back to groups of experts at all, not even small groups. The genuine experts who dissent from the consensus may not constitute a group at all, inasmuch as they share little in the way of common claims, hypotheses or approaches (Nuccitelli 2015; Lewandowsky, Cook & Lloyd in press).

12 This result is congruent with formal demonstrations that biased assimilation may be rational, even when it leads to belief polarization (Jern et al.; Cook and Lewandowsky 2016 demonstrate that the actual belief update of climate ‘sceptics’ may be consistent with Bayesian principles, given plausible assumptions). Both models assume that differential weight is given to the 2014 testimony of sources; the hypothesis advanced here provides a mechanism for assigning such weight.

13 A reviewer for this journal suggests that in fact dissent from the expert consensus may be common and uncorrelated with political allegiance. There is evidence that acceptance of conspiratorial thinking is very common and cuts across political lines (Uscinski and Parent 2014). Such dissent may be enabled by epistemic individualism. However, there does appear to be a conspiratorial disposition, associated with an endorsement of multiple conspiracy theories and also with a rejection of scientific consensus (Lewandowsky et al. 2013a). We may all accept a conspiracy theory, but we are not all given to conspiratorial ideation Conspiratorial ideation and endorsement of free-market ideology independently predict rejection of the scientific consensus; the second more strongly than the first (Lewandowsky et al. 2013a).

14 I thank a reviewer for drawing my attention to Betz et al. (2013), which shows that under some circumstances a community may identify truths more effectively by treating all agents as equally knowledgeable, even when they are able to come to identify genuine expertise. Identifying the circumstances in which differential deference would outperform such equal time deference would require detailed modelling. The wide range of evidence that we do in fact defer differentially suggests that nevertheless on average such deference has been adaptive in ancestral environments and continues to be so today.
